# Book Review

**Published:** 1912-06

**Authors:** 


**Bulletin of the Committee for the Study of Special Diseases.** Edited by T. S. P. Strangeways, M.A., M.R.C.S. Vol. III. Published by the Committee. 1910–11.—This report is not equal in importance and interest to those which have preceded it. It is chiefly occupied by a comparison of the metabolic processes, and particularly the uric acid excretion in gout and rheumatoid arthritis. The reports show that although some few cases of rheumatoid arthritis show a prolongation in the time taken for the elimination of exogenous nitrogen, yet the great mass of osteo-arthritic cases can be sharply differentiated from true gout. The reports on the urine, the pulse rate and the temperature in rheumatoid arthritis will be useful for reference, and bear out the observations of previous writers. The chapters devoted to the association of rheumatoid arthritis and vaginal discharge show that there is some relationship between the presence of the discharge and the onset of the arthritis. It is too of great interest to note that one of the commonest micro-organisms found in this discharge is the pseudo-diphtheria bacillus. Most medical men would confirm this finding, and many have treated the disease with vaccines prepared from pseudo-diphtheria, but with disappointing results. The value of these findings would be much enhanced had we been told whether vaginal discharges were more frequent amongst women suffering from rheumatoid arthritis than amongst those not so afflicted, and whether the pseudo-diphtheria bacillus was equally common in vaginal discharges occurring in patients suffering from other diseases. The work of this Committee is hampered by its lack of funds, and owing to this reason it had to close its Research Hospital in 1908. A special appeal to medical men was made which, although not bringing in anything approaching the sum of money required, enabled them to continue their work at a later date. The Committee are anxious to place their work on an assured financial basis.

**Manual of Surgery.** By Alexis Thomson and Alexander Miles. Fourth Edition. Vol. I. Pp. xix, 809. London: Oxford Medical Publications. 1911.—This excellent text-book for students and for reference is well known as in the front rank both for teaching students and as a book of reference. In the fourth edition just published the authors have brought the manual well up to date. An excellent account is given of modern advances in the diagnosis and treatment of syphilis. Some new illustrations are added, and more attention is given to surgical anatomy. The writers propose to add a third volume dealing with operative surgery. More details of method might well be given, as for instance with reference to the use of carbon dioxide snow, aneurysmorraphy, and other procedures, the magnesium sulphate treatment of tetanus should be mentioned, and the calcium lactate for chilblains.

**A Synopsis of Surgery.** By Ernest W. Hey Groves, M.S., M.D. Third Edition. Pp. viii., 585. Bristol: John Wright & Sons Ltd. 1911.—This edition has followed close on the last, and contains a good many alterations, including some illustrations of surface markings, which in spite of the necessary condensation give the important points very well. The book is useful to students as a dress rehearsal before examinations, and to the practitioner in quickly looking up the chief points of a case. It should also prove an aid to teachers in revising a subject before taking a class.

**Clinical Surgery.** By C. B. Lockwood. Second Edition. Pp. viii., 349. London: Oxford Medical Publications. 1911. —We are most pleased to welcome a second edition of this very useful collection of clinical lectures and addresses by Mr. Lockwood. We are glad, too, to notice that the scope of the work is being widened, two new chapters being added in this edition upon "Fractures of the Patella and their Surgical Treatment," and upon "Amputations at the Hip and Shoulder." We venture to think that Mr. Lockwood's method of always using annealed silver wire would probably be changed for kangaroo or reindeer tendon if he gave it a more extended trial for bringing together the fragments in fractured patella. We are ourselves inclined, too, to look upon the routine use of a drainage tube in these operations as a danger rather than a safeguard, and regard the position of the limb with efficient firm pressure as sufficient means to prevent fluids collecting in the synovial pouches. Mr. Lockwood's style is delightful, and to read his book affords us both pleasure and valuable instruction.

**Chirurgisches Vademecum für den praktischen Arzt.** Von Professor Dr. Alfred Schönwerth. Pp. xi., 167. Munich: Lehmann. 1912.—In this little book, which is clearly printed and well illustrated, the author has managed to say a great deal about all that is really essential in cases of surgical emergency in an incredibly short space. Practically every condition which may result from accident or infection, or otherwise constitute a surgical emergency, is dealt with in a brief and precise manner. There can be no doubt that the author has achieved a veritable triumph in his combination of brevity with lucidity.

**Surgical Applied Anatomy.** By Sir FREDERICK TREVES, Bart., G.C.V.O., C.B., LL.D., F.R.C.S. Sixth Edition. Revised by ARTHUR KEITH, M.D., LL.D. Aber., F.R.C.S. Pp. 676. London: Cassell & Co. 1911.—This manual for students of medicine is an old friend now brought up to date. The text is clearly written, the diagrams are excellent, and the index comprehensive. A most useful book for all students of surgery, and especially so for those reading for their final examinations.

**First-Aid Charts.** Compiled by EDWARD C. B. IBOTSON, M.B., B.S. London. Bristol: John Wright & Sons Ltd.—We have received from the publishers four large cards about two feet square suitable for hanging on the walls of lecture rooms, studies, factories and ambulance stations, containing the necessary details for the proper rendering of First Aid. These Charts are clearly printed, well arranged and simple in language. It is easy to see at a glance what to do in any case of emergency. The first table deals with emergencies, accidents and poisons. The second explains fractures, dislocations and sprains—giving the names of the bones, the causes and recognition of the injury, with the First-Aid treatment. The third treats of hemorrhage and wounds, and the fourth of insensibility from accident, poison and fits. The whole subject-matter is admirably worked out by the compiler, Dr. Edward C. B. Ibotson, and when, as at present, there is such a demand for learning First-Aid work in all the voluntary aid detachments and other ambulance classes, the lecturer and the student will find these Charts of the greatest assistance.

**Practical Anæsthetics.** By H. EDMUND G. BOYLE, M.R.C.S., L.R.C.P. Second Edition. Pp. x., 209. London: Oxford Medical Publications. 1911.—Mr. Boyle is to be congratulated on having kept strictly within the limits of practical anæsthesia. The first edition was deservedly very popular, and we are convinced that a similar appreciation will be extended toward the present edition. Almost every sentence in the book contains some essential detail of instruction in the art of anæsthetising. The lists of indications and contra-indications for guidance in the choice of anæsthetic may appear to be somewhat dogmatic, but cannot fail to give the reader a very clear idea as to how to select the most appropriate method for each case. This clearness, which obtains throughout the work, has enabled the author to produce a very useful chapter on sequel of anæsthetics, with concise and definite rules as to when to substitute one anæsthetic for another. Spinal analgesia is dismissed with such brevity, that one is led to suppose that the author does not make very extensive use of this method.

